# Revisiting the identification of tumor sub-volumes predictive of residual uptake after (chemo)radiotherapy: influence of segmentation methods on ^18^F-FDG PET/CT images

**DOI:** 10.1038/s41598-019-51096-x

**Published:** 2019-10-17

**Authors:** Mathieu Hatt, Florent Tixier, Marie-Charlotte Desseroit, Bogdan Badic, Baptiste Laurent, Dimitris Visvikis, Catherine Cheze Le Rest

**Affiliations:** 1 LaTIM, INSERM, UMR 1101, Univ Brest, Brest, France; 2 Nuclear Medicine department, CHU Milétrie, Poitiers, France

**Keywords:** Cancer imaging, Biomedical engineering, Computer science

## Abstract

Our aim was to evaluate the impact of the accuracy of image segmentation techniques on establishing an overlap between pre-treatment and post-treatment functional tumour volumes in ^18^FDG-PET/CT imaging. Simulated images and a clinical cohort were considered. Three different configurations (large, small or non-existent overlap) of a single simulated example was used to elucidate the behaviour of each approach. Fifty-four oesophageal and head and neck (H&N) cancer patients treated with radiochemotherapy with both pre- and post-treatment PET/CT scans were retrospectively analysed. Images were registered and volumes were determined using combinations of thresholds and the fuzzy locally adaptive Bayesian (FLAB) algorithm. Four overlap metrics were calculated. The simulations showed that thresholds lead to biased overlap estimation and that accurate metrics are obtained despite spatially inaccurate volumes. In the clinical dataset, only 17 patients exhibited residual uptake smaller than the pre-treatment volume. Overlaps obtained with FLAB were consistently moderate for esophageal and low for H&N cases across all metrics. Overlaps obtained using threshold combinations varied greatly depending on thresholds and metrics. In both cases overlaps were variable across patients. Our findings do not support optimisation of radiotherapy planning based on pre-treatment ^18^FDG-PET/CT image definition of high-uptake sub-volumes. Combinations of thresholds may have led to overestimation of overlaps in previous studies.

## Introduction

Multi-modality ^18^FDG-PET/CT is the most used imaging method in the diagnosis and outcome monitoring for head and neck (H&N) and esophageal cancer patients. Despite the interest for the use other PET tracers^1,2^, these ^18^FDG images are being predominantly exploited for radiotherapy treatment planning purposes and for assessment of response to treatment. Previous studies have investigated the possibility of exploiting pre-treatment ^18^FDG PET/CT images to predict the spatial location and size of residual or relapsed lesions^3–11^. The main hypothesis behind these studies is that residual/relapse uptake volumes (as seen on post-treatment PET/CT images) mainly correspond with tumour higher uptake sub-volumes identified on pre-treatment PET/CT images. If confirmed, this could support a radiotherapy planning optimisation strategy based on increasing the dose to these identified tumour sub-volumes on pre-treatment images^12^. Within this context, proof-of-concept studies in lung cancer patients suggested that a 50% SUV_max_ threshold-based sub-volume on pre-treatment PET image corresponded well with the residual uptake (defined with a 70% SUV_max_ threshold), according to the metric overlap fraction (OF)^3,4^. Subsequently, two other studies showed similar results in lung cancer^5,7^. Similar findings were also obtained in 24 rectal cancer patients^6^ using the same approach, although the use of deformable image registration to align the pre- and post-treatment PET/CT images, is likely to have biased the overlap analysis by deforming overall tumour volumes. Finally, more recently, four other studies have exploited several combinations of threshold values and additional overlap metrics in 17 non-small-cell lung cancer (NSCLC)^8^, 32 esophageal^9^, and 19^10^ or 38 H&N^11^ cancer patients, with variable results. The obtained overlaps in oesophageal and NSCLC patients were estimated to be sufficiently high to justify radiotherapy planning optimisation, whereas in H&N cancer patients the overlaps were lower, which was attributed to registration and positioning/lack of contention issues^10,11^.

Most of these previous studies exploited arbitrary values of %SUV_max_ image thresholds, despite the well-established limited accuracy and robustness of such methodology for the segmentation of PET image based functional volumes^13–16^. Indeed, optimal threshold values greatly depend on lesion size and contrast^17^, in addition to being very challenging to determine in case of high intratumor uptake heterogeneity where relationship between maximum intensity and overall contrast can be quite different than for homogeneous lesions. A recent MICCAI challenge further highlighted these limitations, as both implemented fixed thresholds (40 and 50% of the maximum) were amongst the worst ranked methods^18^. Additionally, an “optimal” deterministic threshold may never be found for specific cases, for example with limited signal-to-noise ratio, for which only additional criteria (*e*.*g*., spatial relationships between voxels) have the potential to provide a satisfactory segmentation result. It is thus difficult to evaluate whether positive or negative results are due to the choice of inappropriate and arbitrary threshold values and/or inappropriate overlap metrics, or the result of actual imperfect biological overlap between pre- and post-treatment activity distributions. Based on accuracy results shown by previous studies using these thresholding approaches for functional volume segmentation, one can assume that their use may lead to inaccurate volume segmentations and associated biases in the overlap estimates, especially when the true underlying overlap is small or non-existent. An alternative approach would be the use of more accurate and robust segmentation algorithms for the definition of both the pre- and post-treatment PET image tumour volumes, in an attempt to extract more reliable overlap estimates and provide a more robust and accurate answer to the initial hypothesis. The main objective of this study was therefore to compare previously considered image threshold combinations with one, amongst others, accurate and robust automatic method able to define simultaneously a pre-treatment high uptake sub-volume (denoted from here onwards as V1 from PET1) and a post-treatment residual uptake (denoted from here onwards as V2 from PET2). We used both a realistic simulated tumour example with known ground truth in order to better elucidate the behaviour of each of the approaches considered, while a cohort of both oesophageal and H&N cancer patients was used to provide a clinically relevant assessment.

## Materials and Methods

One example case consisting of simulated PET images with known ground-truth was initially used to elucidate the behaviour of the combinations of threshold values most often previously considered in the literature, with respect to three different known overlap configurations. In addition, we retrospectively collected pre- and post-treatment ^18^FDG-PET/CT scans of patients with oesophageal and H&N cancer treated by (chemo)radiotherapy. We identified those presenting residual uptake in PET2 and co-registered PET1 and PET2 images using rigid transformations to avoid any deformation and intensity biases associated with non-rigid deformations. The pre-treatment tumour volume and V1, as well as V2 were subsequently delineated using either combinations of threshold values, or the advanced method. The determined spatial overlap between V1 and V2 was quantified using four metrics also used in most previous studies.

### Simulated images

PET images of the simulated example were obtained according to a previously described workflow using the Geant4 Application for Tomographic Emission (GATE) version 6.0^19,20^. For the attenuation map, the tumour in the simulations were considered as soft tissue and the background as lung. Two minutes acquisitions simulated in a model of the Siemens Biograph-6 were reconstructed with OSEM (3 iterations, 21 subsets) using the CASToR software (Customisable and Advanced Software for Tomographic Reconstruction, http://www.castor-project.org)^21^ and post-filtered with a 5 mm 3D Gaussian. Voxel size of both the ground-truth map and the corresponding reconstructed images was 4 × 4 × 4 mm^3^. Parameters were chosen to be similar as the clinical data used in the present work (although acquired in a more recent scanner). Additional details can be found in^20,21^.

**Figure 1:**
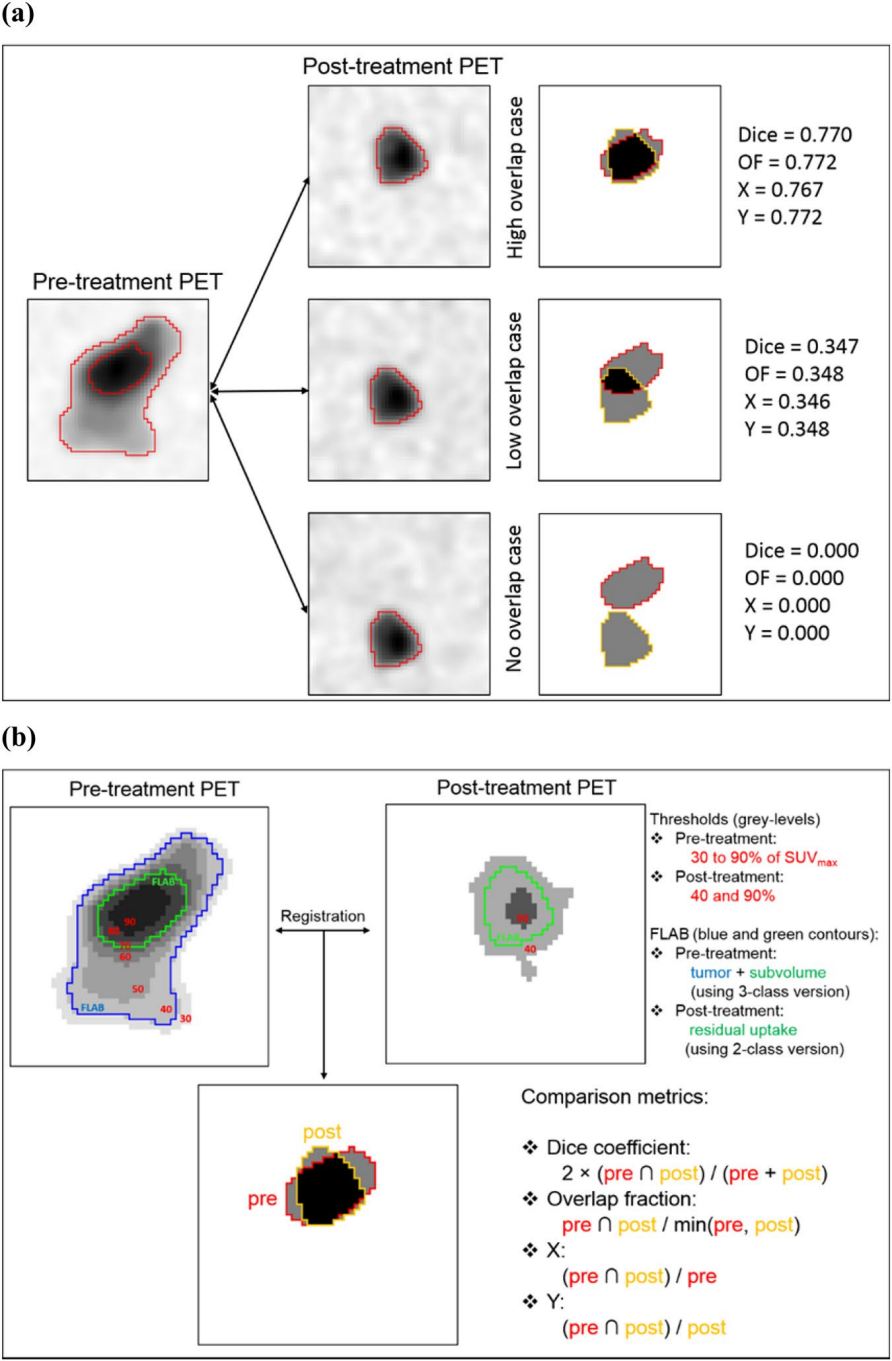
(**a**) Simulated images for the toy example. On the left images, red contours are ground-truth. On the overlap maps (right), red and orange contours are V1 and V2 respectively. The black area identifies the true overlap between V1 and V2. (**b**) Analysis workflow illustrated on the toy example. PET1 and PET2 are co-registered. In PET1 the thresholds between 30% and 90% of SUV_max_ are applied, as well as the FLAB algorithm with 3 classes (blue and green contours). In PET2, two thresholds (40% and 90% of SUV_max_) and the FLAB algorithm with two classes (green contour) are used.

In PET1, the tumour was simulated with heterogeneous uptake using a measured contrast between the entire tumour and background of 3:1, whereas V1 included approximately 20% of the entire tumour and was set at a contrast of 2:1 with the rest of the tumour (resulting in a contrast of 6:1 relative to the background). In order to simulate reduced uptake for the residual in PET2, V2 was simulated as a more homogeneous, but smaller (about the same size as V1) and with lower contrast (2:1) with respect to the background. Three configurations were simulated by translating the V2 ground-truth within PET2: the first with a substantial although imperfect overlap (*i*.*e*., not exactly the same location/size/shape, all four overlap metrics of approximately 0.77), the second with a small overlap (all metrics of approximately 0.35), and the last with non-existent overlap (all metrics at 0) (Fig. 1a). These variable overlaps could arise from imperfect (or lack of) biological correlates, registration issues, or a combination of both. Note that for each overlap configuration, all 4 metrics provide very close and consistent values (Table 1).

**Table 1:** Results of the simulation study.

		GT	FLAB	3040	4040	5040	6040	7040	8040	9040	3090	4090	5090	6090	7090	8090	9090
High true overlap																	
	Dice	0.770	0.788 (+2%)	0.488 (−37%)	0.562 (−27%)	0.668 (−13%)	0.763 (−1%)	0.763 (−1%)	0.677 (−12%)	0.415 (−46%)	0.073 (−91%)	0.092 (−88%)	0.129 (−83%)	0.191 (−75%)	0.260 (−66%)	0.355 (−54%)	0.587 (−24%)
	OF	0.772	0.869 (+13%)	0.979 (−27%)	0.948 (+22%)	0.886 (+15%)	0.793 (+3%)	0.882 (+14%)	0.980 (+27%)	1.000 (+30%)	1.000 (+30%)	1.000 (+30%)	1.000 (+30%)	1.000 (+30%)	1.000 (+30%)	1.000 (+30%)	0.970 (+26%)
	X	0.767	0.720 (+6%)	0.325 (−58%)	0.400 (−48%)	0.537 (−30%)	0.735 (−4%)	0.882 (+15%)	0.980 (+28%)	1.000 (+30%)	0.038 (−95%)	0.048 (−94%)	0.069 (−91%)	0.105 (−86%)	0.149 (−81%)	0.216 (−72%)	0.421 (−45%)
	Y	0.772	0.869 (+13%)	0.979 (+27%)	0.948 (+23%)	0.886 (+15%)	0.793 (+3%)	0.672 (−13%)	0.517 (−33%)	0.262 (−66%)	1.000 (+30%)	1.000 (+30%)	1.000 (+30%)	1.000 (+30%)	1.000 (+30%)	1.000 (+30%)	0.970 (+26%)
	Acc	N/A	0.960	0.715	0.722	0.737	0.765	0.813	0.877	0.811	0.635	0.635	0.635	0.635	0.635	0.635	0.631
Low true overlap																	
	Dice	0.347	0.385 (+11%)	0.519 (+50%)	0.594 (+71%)	0.690 (+99%)	0.562 (+62%)	0.516 (+49%)	0.440 (+27%)	0.309 (−11%)	0.079 (−77%)	0.099 (−71%)	0.140 (−60%)	0.178 (−49%)	0.156 (−55%)	0.355 (−54%)	0.036 (−90%)
	OF	0.348	0.422 (+21%)	1.000 (+187%)	0.964 (+177%)	0.886 (+154%)	0.569 (+63%)	0.615 (+77%)	0.660 (+90%)	0.776 (+123%)	1.000 (+187%)	1.000 (+187%)	1.000 (+187%)	0.861 (+147%)	0.556 (+60%)	0.222 (−36%)	0.056 (−84%)
	X	0.346	0.354 (+2%)	0.351 (+1%)	0.429 (+24%)	0.566 (+64%)	0.556 (+61%)	0.615 (+78%)	0.660 (+91%)	0.776 (+124%)	0.041 (−88%)	0.052 (−84%)	0.075 (−78%)	0.099 (−71%)	0.090 (−74%)	0.216 (−72%)	0.026 (−92%)
	Y	0.348	0.422 (+21%)	1.000 (+187%)	0.964 (+177%)	0.886 (+154%)	0.569 (+63%)	0.444 (+28%)	0.330 (−5%)	0.193 (−45%)	1.000 (+187%)	1.000 (+187%)	1.000 (+187%)	0.861 (+147%)	0.556 (+60%)	0.222 (−36%)	0.056 (−84%)
	Acc	N/A	0.858	0.59	0.593	0.601	0.658	0.702	0.772	0.545	0.184	0.184	0.184	0.202	0.273	0.573	0.518
No true overlap																	
	Dice	0	0	0.445	0.470	0.363	0.141	0.087	0.028	0	0.075	0.094	0.101	0	0	0	0
	OF	0	0	0.911	0.809	0.489	0.149	0.1	0.039	0	1	1	0.765	0	0	0	0
	X	0	0	0.294	0.331	0.288	0.134	0.1	0.039	0	0.039	0.049	0.054	0	0	0	0
	Y	0	0	0.911	0.809	0.489	0.149	0.078	0.021	0	1	1	0.765	0	0	0	0

### Patients PET/CT datasets

Fifty-four patients treated in the University Hospital of Poitiers, France, were retrospectively included. All patients had locally advanced disease treated by combined radiochemotherapy and provided signed permission for the use of their clinical data for scientific purposes and informed consent for the anonymous publication of data. The “comité de protection des personnes (CPP Ouest III)” (ethics committee) from the University Hospital of Poitiers approved this study. Each patient underwent both a pre-treatment and a post-treatment ^18^FDG-PET/CT scan 3 months after the end of treatment, in the same PET/CT scanner (Biograph mCT 40 ToF with axial field of view of 21.6 cm, Siemens, Erlangen, Germany) using a routine clinical protocol. PET/CT acquisition began after 6 hours of fasting and 60 ± 5 min after injection of 2.5 MBq/kg of ^18^F-FDG (421 ± 98 MBq, range 220–695 MBq). Non-contrast enhanced, non-respiratory gated (free breathing) CT images were acquired (120 kVp, Care Dose® current modulation system) with an in-plane resolution of 0.853 × 0.853 mm^2^ and a 5 mm slice thickness. PET data were acquired using 3.5 minutes per bed position and images were reconstructed using a CT-based attenuation correction and the OSEM-TrueX-TOF algorithm (with time-of-flight and spatial resolution modelling, 3 iterations and 21 subsets, 5 mm 3D Gaussian post-filtering, voxel size 4 × 4 × 4 mm^3^).

Thirty-seven patients (17 with oesophageal, either adenocarcinoma (25%) or squamous cell carcinoma stage (75%), and 20 with H&N squamous cell carcinoma stage IV tumours) out of 54 (69%) had a residual uptake in PET2 and were thus further analysed (registration and overlap determination).

PET1 and PET2 for these 37 patients were automatically registered with a rigid body transformation determined on the CT datasets, using the 3D Slicer^TM^ Expert Automated Registration module^22^ optimised with the Mattes mutual information metric^23^. In order to simplify the registration, only the body region containing the tumour was used. The transformation was initialised with a registration of the two centres of mass of the images. The obtained transformation was then applied to the corresponding PET.

### V1 and V2 determination

For the automatic approach, we chose the Fuzzy Locally Adaptive Bayesian (FLAB) algorithm because of its capability to automatically determine both the entire volume and the high uptake sub-volume in the pre-treatment image^24^. In contrast to threshold-based segmentation, FLAB relies on an estimation of the contrast and properties of statistical distributions, as well as on spatial correlation between voxels to classify each voxel^24,25^. Regarding the combination of thresholds, we replicated the configurations of the majority of the most recent studies^8–11^.

Therefore on PET1, eight different V1 were determined using 30%, 40%, 50%, 60%, 70%, 80% and 90% of SUV_max_^3,4,8–11^ and FLAB (Fig. 1b). In order to define V2 in PET2, 3 different volumes were obtained with thresholds at 40% and 90%^8,9,11^ or FLAB (Fig. 1b). FLAB was applied in PET1 using 3 classes (one for background and two for tumour) to define an overall tumour volume and V1 (the high-uptake sub-volume)^24^. In PET2, FLAB was applied with 2 classes^25^ in order to define V2.

We emphasise that when V2 is measured as larger than V1, carrying out the overlap analysis is irrelevant. In these cases, the “residual” volume would likely encompass a large part of the (or even the entire) pre-treatment volume and very likely the entire high uptake sub-volume, leading to biased overlap metrics. It would also be meaningless to use such candidate sub-volume in PET for dose boosting, since it would mean either boosting the entire pre-treatment volume or a sub-volume of it that would not cover the entire residual volume (V2). The overlap analysis was thus carried out only for the cases with V2 < V1 according to the *a priori* more accurate delineation approach (*i*.*e*., FLAB).

### Overlap analysis

The overlap between V1 and V2 was quantified with 4 metrics^8–11^ (see Fig. 1b). These included Dice coefficient, overlap fraction (OF), and intersection of V1 and V2 volumes divided by either V1 (X) or V2 (Y). Jaccard coefficients were not used as they provide redundant ranking with Dice.

$$Dice=2\times \frac{{V}_{1}{\cap }^{}{V}_{2}}{{V}_{1}+{V}_{2}},\,\,\,OF\,\frac{{V}_{1}{\cap }^{}{V}_{2}}{min({V}_{1},{V}_{2)}},\,\,\,X=\frac{{V}_{1}{\cap }^{}{V}_{2}}{{V}_{1}}\,{\rm{and}}\,\frac{{V}_{1}{\cap }^{}{V}_{2}}{{V}_{2}}.$$

Dice coefficients are sensitive to the differences in the size of the two compared volumes, whereas OF leads to higher values due to the use of the smallest volume in the denominator. In other words it is possible to achieve “correct” values for these metrics even if the compared volumes are not accurate and their overlap is not spatially (in terms of absolute volume or location/shape) correct. In the clinical data where no ground-truth is available, only these metrics were calculated. For the simulated cases on the other hand, they were complemented with an accuracy estimation of the overlap determined through the segmentation methods quantified by the mean of positive predictive value (PPV) and sensitivity (SE)^13^.

All combinations of threshold values (between 30% and 90% for V1, combined with either 40% or 90% for V2) were analysed and will be denoted in the following as *xxyy*, were *xx* is 30, 40, 50, 60, 70, 80 or 90 (V1) and *yy* is 40 or 90 (V2). For FLAB, the overlap was quantified between the high-uptake sub-volume in PET1 and the residual/relapse uptake in PET2 (Fig. 1b). Statistical comparison between the metrics distributions were carried out using rank Mann-Whitney tests. P < 0.01 was considered significant. Statistics are reported in the text as mean ± standard deviation (median).

### Approval, accordance and informed consent

The “comité de protection des personnes (CPP Ouest III)” (ethics committee) from the University Hospital of Poitiers approved this study. All procedures performed in studies involving human participants were in accordance with the ethical standards of the institutional and/or national research committee and with the 1964 Helsinki declaration and its later amendments or comparable ethical standards. Informed consent was obtained from all individual participants included in the study.

## Results

Table 1 provides the analysis results of the simulated cases both qualitatively (visually) and quantitatively (Dice, OF, X, Y and the overlap accuracy). None of the threshold configurations provided satisfactory results across all situations. This was mostly due to inaccurate segmentations in PET2 (V2 too small for 90% and too large for 40%). In PET1, 60–80% thresholds provided segmentations close to the FLAB high-uptake sub-volume (as shown in Fig. 1b). 6040 and 7040 provided even better estimates of the overlap metrics (−1% to +3% for 6040, −1% to +15% for 7040) than FLAB in the case of substantial overlap. However, they did so through less accurate overlap determination (accuracy of 0.765 and 0.813 respectively) and they led to large overestimation for small overlap cases (+50% to +80%). In addition, they detected an overlap (although small) for the case with non-existent overlap in the ground-truth. Overall, all threshold combinations were found to either strongly over- or under-estimate the true overlap metrics, as well as the true overlap spatial location and size (mean accuracy of 0.706 ± 0.084 and 0.470 ± 0.215 for high and low overlap respectively). In particular, there was a trend in overestimating the overlap in cases where it is actually small or non-existent. In contrast, FLAB provided estimates of the overlap metrics between +2 and +21% for the high and small overlap cases and was also capable of accurately detecting the lack of overlap. More importantly, it provided more accurate estimation of the true overlap spatial extent and localisation (accuracy of 0.960 and 0.858 for the high and the small overlap cases respectively, *vs*. 0.877 and 0.772 obtained for the best threshold combination of 8040).

**Figure 2:**
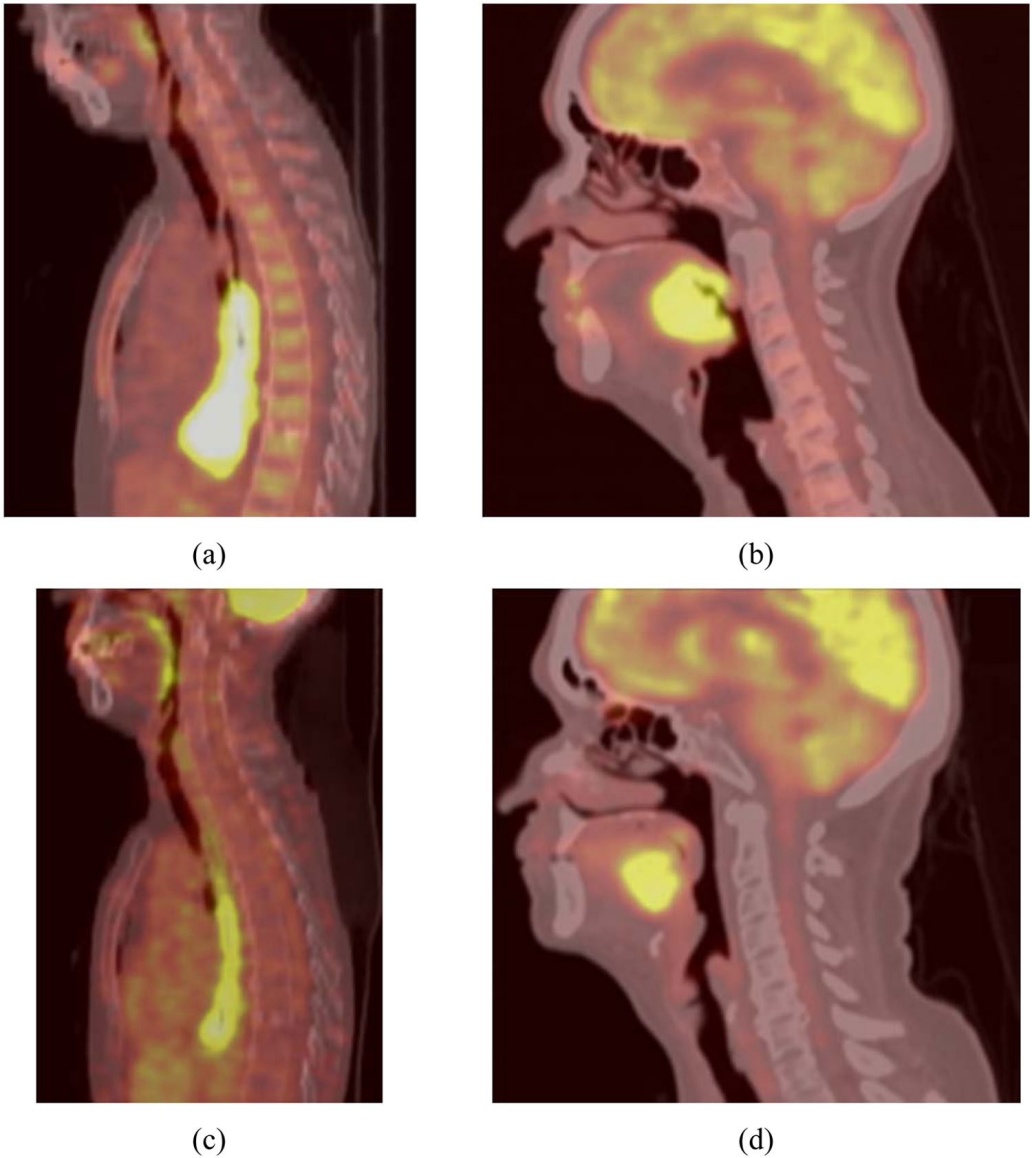
Examples of (**a**,**c**) pre-treatment and (**b**,**d**) post-treatment PET/CT images in (**a**,**b**) esophageal and (**c**,**d**) H&N tumors.

In the clinical cases, histology and stage had no significant impact on SUV measurements. Registration was easier to perform and led to more visually accurate results for oesophageal cancer cases compared to H&N, mostly as a result of the large differences in the position of the patients’ heads between PET1 and PET2, due to the lack of using immobilisation devices or positioning standardisation protocols (see Fig. 2).

**Figure 3:**
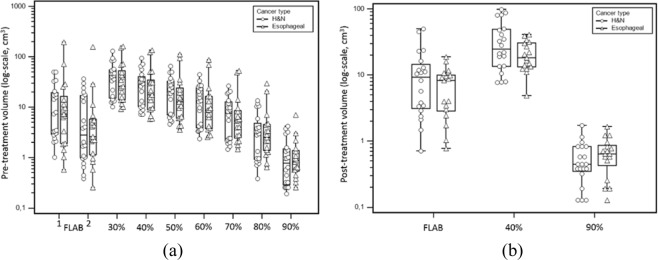
Measurements of (**a**) pre-treatment volumes in PET1 and (**b**) relapse/residual uptakes (V2) in post-treatment PET images, using FLAB and thresholds. In (**a**), for FLAB, ‘1’ denotes the entire uptake volume, whereas ‘2’ denotes the high-uptake sub-volumes (i.e., V1). Note the difference of scale in the y axis between (**a**,**b**).

In the absence of ground-truth and based on previous results and those of the simulated example in this study, FLAB was assumed to provide more accurate and robust volumes compared to fixed thresholds^18^. According to FLAB, the entire tumour volumes in PET1 were 23.4 ± 47.8 (7.3) cm^3^ for oesophageal tumours and 14.7 ± 15.8 (7.7) cm^3^ for H&N tumours, with significantly smaller (p < 0.01) high-uptake sub-volumes (V1) of 13.9 ± 37.8 (2.6) and 9.1 ± 11.2 (2.9) for oesophageal and H&N respectively. By comparison, V2 in PET2 were measured as 7.3 ± 5.3 (8.3) cm^3^ (oesophageal) and 12.5 ± 13.8 (9.3) cm^3^ (H&N) (Table 1, Fig. 3). Overall, reduction of uptake volume in oesophageal tumours (−58 ± 32%) was observed for only 9/17 patients, with volume increase (+148 ± 150%) for the other 8. For H&N, a reduction was similarly observed only in 10 patients (−47 ± 29%), whereas for the other half, an increase of volume was measured (+159 ± 281%).

As shown in Fig. 3, FLAB provided volumes that were significantly different than most threshold-based segmentations. Compared to FLAB-defined V1 (Fig. 3a), those obtained with thresholds showed tighter spread and larger values with thresholds between 30% and 60% (p < 0.001 for 60%, p < 0.0001 for 30% to 50%), smaller values with 90% (p < 0.001), and values in a similar (not statistically different) range for 70% and 80% (p = 0.06 and 0.54 respectively). Similarly, compared to FLAB, V2 defined with both 40% and 90% (Fig. 3b) were significantly different (p < 0.0001). A 40% threshold led to larger V2 of 21.4 ± 10.7 (18.2) cm^3^ for oesophageal tumours and 36.4 ± 29.2 (25.1) cm^3^ for H&N tumours, whereas a 90% threshold led to smaller V2 of 0.7 ± 0.4 (0.6) cm^3^ for oesophageal tumours and 0.6 ± 0.4 (0.5) cm^3^ for H&N tumours (Fig. 3b). It is worth noting that the use of threshold combinations led to variable numbers of patients with V2 < V1: for xx90 all V2 were smaller than V1 except 8090 (n = 35) and 9090 (n = 21). For xx40, the number was much more variable, between 25 for 3040 to 0 with 9040 (4040: n = 14, 5040 and 6040: n = 8, 7040: n = 4 and 8040: n = 1).

**Figure 4:**
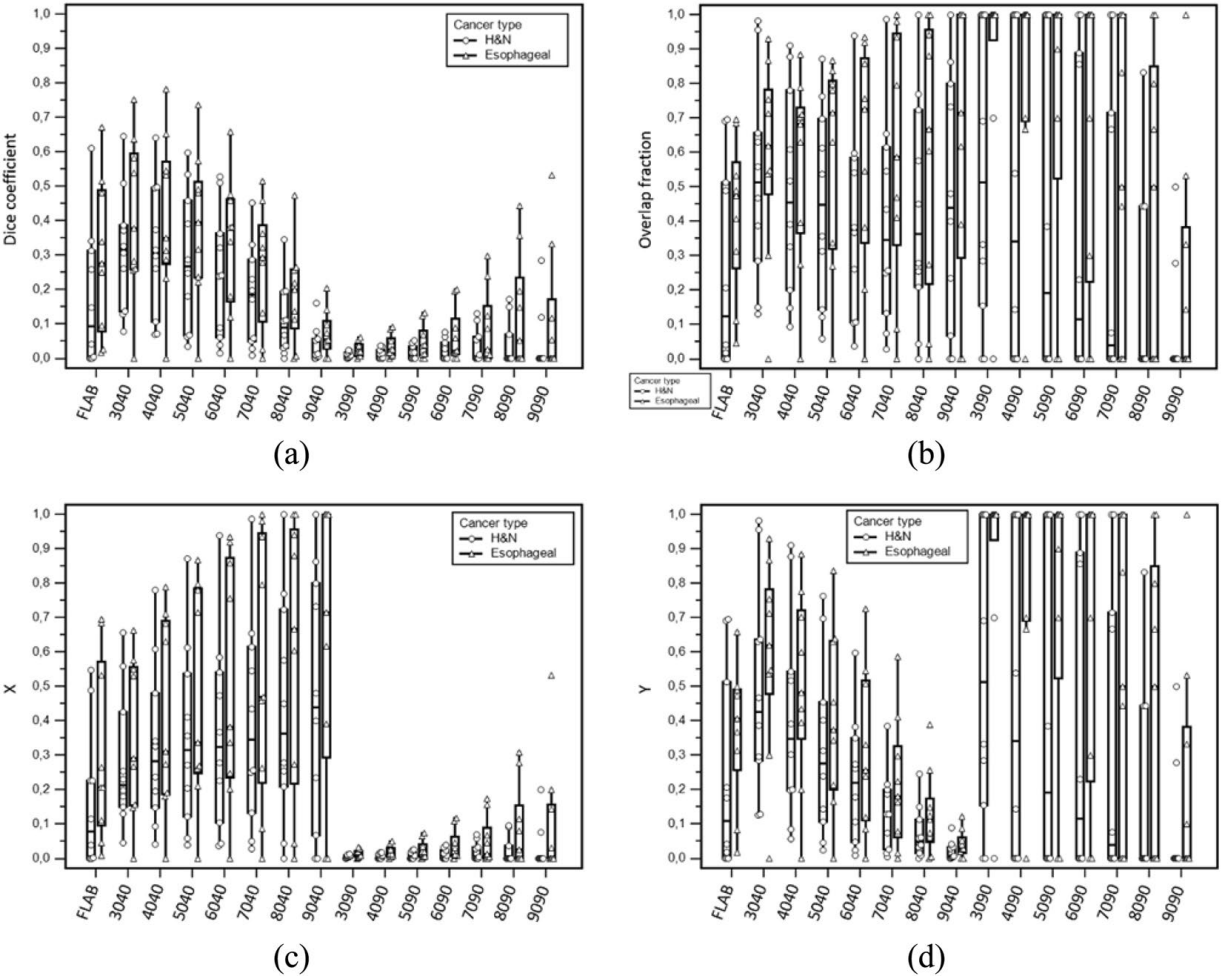
Overlap assessment using (**a**) Dice, (**b**) OF, (**c**) X and (**d**) Y.

The subsequent overlap analysis was carried out for the 9 oesophageal and the 10 H&N cases for which V2 < V1 according to FLAB. Table 1 in the supplemental material provide statistics for each overlap metric and for each segmentation combinations whereas Fig. 4 provides graphs with raw data. Agreement between V1 and V2 were consistently higher for oesophageal cases compared to H&N, across all metrics and for both the threshold-based and FLAB comparisons, even though the differences were not statistically significant due to the very large standard deviation of all distributions (p > 0.1) (Fig. 4). Indeed, they were highly variable across patients, in both pathologies and whatever overlap metric was considered, or how V1 and V2 were defined, with values as low as 0, and some reaching 1. The most consistent results across metrics were obtained with FLAB, which identified low to moderate agreement ( < 0.7), independently of the overlap metric considered, in contrast to the various combinations of threshold that exhibited highly different behaviour depending on the chosen metric and the combination of threshold used.

Dice index led to the lowest agreements, whereas OF led to the highest. X highlighted higher agreement when considering xx40 combinations compared to very low agreement when considering xx90 combinations, whereas it was the opposite for Y that led to higher overlaps for xx90 configurations compared to xx40 ones. By contrast, FLAB led to more similar (and moderate) results between X and Y. For most metrics and configurations, a large variability across patients was noted.

## Discussion

The results using a simple simulated example with 3 different overlap configurations allowed to elucidate that none of the threshold combinations most often considered in recently published studies were able to provide satisfactory overlap determination and associated metrics measurements for all configurations, in contrast to an advanced method, such as FLAB considered in this study. Although, this behaviour was observed in a single and simple simulated example, it illustrates well the expected behaviour of the various threshold combinations. The main conclusions are that i) accurate estimation of overlap metrics (i.e., Dice, OF, X, Y) can arise from inaccurate segmentations and thus inaccurate determination of the spatial extent (location and size) of the true overlap; ii) these inaccurate threshold combinations can lead to large underestimation of overlaps, but also and perhaps more importantly to large overestimation, especially in cases where the true overlap is small or non-existent. One key point is the accurate determination of V2, for which both 40% (overestimation) and 90% (underestimation) failed in our simulated case. In contrast, a 70% threshold to determine V2^3,4^ leads to a segmentation volume close to the one obtained with FLAB (between 14% and 4% difference across the three images, results not shown) in that specific simulated case. Therefore, the 7070 or 8070 configurations would provide results close to those obtained with FLAB. However, one needs to note that a different contrast and/or tumour size configuration for V2 would obviously require a different threshold value in order to get a result similar to FLAB, since a 70% threshold is not appropriate for all combinations of lesion sizes and contrast. Therefore, even if the 7070 or 8070 configurations would appear to be the best in our simulated tumour example, they would fail to provide consistent results in the clinical cases given their large variability in size and contrast in PET2, as manifested by the clinical cohort used in this study.

The results obtained using the clinical datasets (where no ground-truth is known) are similar to those obtained in the simulation study. In both pathologies considered in the patient cohort, V2 obtained using 40% and 90% threshold were significantly larger and smaller respectively than those obtained with FLAB (Fig. 3b) and V1 obtained with 70–80% threshold were close to those obtained with FLAB (high-uptake sub-volume) (Fig. 3a). Similarly to the simulated case results, the threshold combinations led to high variability in the overlap metrics, and sometimes higher than those provided by FLAB (assumed to be more accurate). The three main conclusions of the analysis in the clinical datasets are: i) a residual uptake was visible in only 69% of the initial cohort of patients; ii) for about half of these patients with residual uptake, in both pathologies, this “residual” uptake was actually larger than the pre-treatment one, precluding an overlap analysis relevant for pre-treatment radiotherapy planning optimisation; iii) in the remaining half of the patients (35% of the initial cohort), the overlap between high-uptake sub-volume of PET1 and the residual uptake in PET2 was found to be highly variable between patients, and overall between low and moderate, irrespectively of the segmentation method used to define the functional volumes and the metric used to quantify these overlaps. Although some threshold combinations led to somewhat high overlap values in some cases, one may hypothesise based on the simulation study observations that they may largely be resulting from inaccurate segmentations. Finally, the results were generally better for oesophageal cancer patients due to less challenging registration conditions compared to the H&N cancer patients acquired without any specific positioning protocol, as reported by others before^10,11^.

Our concurrent observations on the simulated and acquired patient datasets do not entirely support the initial hypothesis of identifying reliable sub-volumes in pre-treatment PET images that could constitute regions for radiotherapy planning optimisation strategies, such dose boosting sub-volumes. Although some substantial overlaps were observed for a few patients, other patients showed very low or even null overlaps. Several studies have reported rather optimistic results in a number of pathologies regarding the ability of selecting intensity threshold values to identify reliable post-treatment residual uptakes from pre-treatment PET images (Table 2). Although our cohort was small, most previous investigations were carried out in similarly small groups (between 7 and 38) since only a fraction of patients usually exhibit relapse/residual disease. In addition, in our study we restricted the overlap analysis to patients for which residual uptake was smaller than the pre-treatment one, which resulted in including only half of the patients (19 out of 37) in the actual overlap analysis. In most previous studies, it appears that all patients with relapse/residual uptakes were included in the overlap analysis, but it was not explicitly stated whether they all had residual uptakes smaller than pre-treatment volumes or not. If post-treatment volumes larger than pre-treatment ones were included, it may have biased at least partly the reported overlaps towards “artificially” higher values. For example in our cohort, including all 37 patients led to OF for 7040 of 0.59 ± 0.35 (median 0.61) compared to 0.49 ± 0.34 (median 0.47) when including only the 19 patients with residual uptakes smaller than pre-treatment ones. Note that although most of xx90 configurations led to V2 < V1, it is worth highlighting that xx40 configurations led to V2 > V1 in a larger part of the cohort (all of them with 9040, down to a minimum of 12 with 3040).

**Table 2:** Summary of previous studies.

Study	Cancer type	Total	Number of patients				Registration	Segmentation on PET1	Segmentation on PET2	Overlap metrics	Agreement
			PET2 positive	Excluded		Overlap analysis					
				V2 > V1	Other reasons						
Abramyuk, *et al*.^5^	NSCLC	10	10	2	0	10	None	2 adaptive thresholds (TrueD^®^ and ROVER^®^)	2 adaptive thresholds (TrueD^®^ and ROVER^®^)	Visual/qualitative	Failures/relapses located mainly at primary site with highest uptake
Aerts, *et al*.^3^	NSCLC	55	28	1	5	22	Rigid	Thresholds 34, 40, 50, 60, 70%	Thresholds Residual defined as SUV above aortic arc. Within residual: 70, 80, 90%, >SUV 2.5 and >SUV 5.0.	OF	Good to excellent
Aerts, *et al*.^4^	NSCLC	12	8	0	1	7	Rigid	Thresholds 34, 40, 50, 60, 70%	Thresholds Residual defined as SUV above aortic arc. Within residual: 70, 80, 90%	OF	Good to excellent
van den Bogaard, *et al*.^6^	Rectal	28	24	Unknown	0	24	Rigid (global) followed by elastic (local)	Adaptive threshold (signal-to-background ratio)	Adaptive threshold (signal-to-background ratio)	Voxels of PET1 and PET2 arranged into 10-bin histograms	Good to excellent
Shusharina, *et al*.^7^	NSCLC	61	19	Unknown	2	17		Threshold 50%	Threshold 80%	OF	Good to excellent
Calais, *et al*.^8^	NSCLC	39	17	Unknown	0	17	Rigid	Thresholds 30–90%	Thresholds 40 and 90%	Dice, Jaccard, OF, X, Y	Moderate to good, depending on metric and threshold
Calais, *et al*.^9^	Oesophageal	98	35	Unknown	3	32	Rigid	Thresholds 30–90%	Thresholds 40 and 90%	Dice, Jaccard, OF, X, Y	Moderate to good, depending on metric and threshold
Chaput, *et al*.^10^	H&N	72	19	Unknown	0	19	Rigid	Thresholds 30–90%	Thresholds 40 and 70%	Dice, Jaccard, OF, X, Y	Low
Legot, *et al*.^11^	H&N	94	38	Unknown	0	19	Rigid	Thresholds 30–90%	Thresholds 40 and 90%	Dice, Jaccard, OF, X, Y	Low to moderate
Present study	Oesophageal	28	17	8	0	9	Rigid	Thresholds 30–90%, FLAB (3 classes)	Thresholds 40 and 90%, FLAB (2 classes)	Dice, OF, X, Y	Low to moderate
	H&N	26	20	10	0	10					Low

On the other hand, our conclusions are in line with the most recent studies on H&N cancer patients, where registration issues were similarly too important to allow for high volume overlaps^10,11^. In both studies rigid registration was used instead of elastic models. Such deformable models could potentially improve registration in such challenging cases but it may also lead to deformation of the tumour volumes and thus bias the spatial overlap analysis, as well as a modification of intensities, whose biological significance can be questionable. Prospective studies with acquisition protocols including immobilisation devices ensuring similar positioning of the patients in pre- and post-treatment PET/CT acquisitions should be carried out to provide a more conclusive response. However for oesophageal cancer cases, even if volume overlaps were consistently higher than for H&N patients, we were not able to confirm the optimistic results previously published^9^. Lesions in the oesophagus are obviously subject to motion and associated organ deformation, but they are assumed to be less affected compared to lung lesions, where promising results were obtained by other investigators, even in the absence of respiratory gating^3–5,7^. It should be noted however that most of the early promising results in lung lesions were obtained using the OF metric, which as shown in our study tends to provide the highest values. Other results previously reported in rectal cancer patients, where elastic registration was used, could also have led to overestimated overlaps due to deformation of tumour volumes when matching pre- and post-treatment datasets^6^.

Within this context, our study is the first to compare the various combinations of threshold values most often used in the previous investigations in addition and in comparison to the use of a more robust and accurate segmentation approach. The FLAB method was originally designed to automatically define the overall volume as well as a high-uptake sub-volume using a combination of three classes, based on the statistical properties and relative contrast of voxel distributions as well as their spatial relationship, which was validated using both simulated and clinical datasets^24^. Similarly and perhaps more importantly, the definition of the entire residual uptake in PET2 using the standard 2-class FLAB method ensured a more robust and accurate determination of its spatial extent, given the demonstrated robustness of FLAB for smaller and lower contrast uptake lesions compared to fixed threshold approaches^18,25,26^. By contrast, it should be emphasised that most of the threshold combinations are arbitrary and do not rely on any rationale, hence potentially leading to artificially large (or low) overlap values, as illustrated in our simulated example. In particular, a 90% of SUV_max_ threshold can lead to very small volumes that mostly underestimate the true extent of the residual uptake, whereas a 40% threshold may not be appropriate either, leading to overestimation of the uptake, especially for smaller, low contrast uptakes^13^. Rigorous validation of the initial hypothesis thus clearly requires not only a feasible co-registration of pre- and post-treatment images, but also an accurate and robust definition of the residual uptake in the post-treatment PET images, in order to localise its corresponding sub-volume within the entire tumour volume in the pre-treatment image.

The validation of this hypothesis should therefore rely on reliable, accurate and robust volume definition of these post-treatment uptake regions across all patients included in a study. In that regard, a number of automatic algorithms for PET image segmentation have been developed over the last decade. All of them have the potential to provide more accurate results for V2 compared to fixed thresholds, and some have shown improved accuracy over FLAB which we used in this study, for the specific task of determining the entire tumour uptake^18^. On the other hand, the determination of V1 is more challenging, given that the goal is to determine a high-uptake sub-volume of the tumour and not the entire uptake. The advantage of FLAB for the present work is that it has been developed and rigorously validated specifically for this task^24^. Although other advanced methods^18,27,28^ could be also considered, for the majority of them similar additional parametrization or optimization would need to be carried out in order to determine simultaneously the high uptake sub-volume in addition to the entire tumour volume. For example, one could consider using a fuzzy C-Means^29^ or a Gaussian mixture model^30^ with 3 (or more) clusters/Gaussian distributions, which could likely lead to results close to those obtained in this work with FLAB.

Assuming that FLAB provided reliable residual uptakes measurements, thresholds at 40% and 90% led to significantly larger and smaller measurements respectively, which can be considered erroneous in most cases and would obviously lead to over and under-estimation of true overlaps respectively between pre- and post-treatment uptake distributions. Based on the results obtained for the simulated example and the observed differences in the clinical cohort used in this study, we can postulate that threshold combinations tend to overestimate the quantitative overlaps between pre- and post-treatment functional tumour volumes, especially when the true overlap is small or non-existent. We therefore advocate that combinations of fixed thresholds should not be used to investigate the issue of overlaps between pre- and post-treatment PET volumes, as they seem to add important uncertainties in addition to the underlying biological variability, as well as pre- and post-treatment image registration issues. In order to obtain more reliable estimates of these overlaps, we advocate the use of robust and accurate advanced PET segmentation methods combined with standardised pre- and post-treatment image acquisition protocols.

## Conclusions

The overlaps between pre-treatment high-uptake sub-volumes and residual disease after (chemo)radiotherapy were found to be moderate in oesophageal tumours and low in head and neck tumours. These overlaps were also highly variable amongst patients. Therefore our results do not support optimisation of radiotherapy planning based on pre-treatment PET/CT definition of a high-uptake sub-volume, even in oesophageal cases for which better results were observed. In addition, it should be emphasised that only a small fraction (35% the initial cohort) of patients could potentially benefit from such an optimisation. Considering the comparison between segmentation approaches, our results suggest that the use of combinations of arbitrary intensity thresholds led to the overoptimistic evaluation of overlaps reported in previous studies.

## Supplementary information

Supplemental material

## Data Availability

PET/CT images and simulated images used in this study can be made available on request for specific research purposes.
