# Supplementary material for: Revisiting the identification of tumor sub-volumes predictive of residual uptake after (chemo)radiotherapy: influence of segmentation methods on 18F-FDG PET/CT images
Source: Sci Rep. 2019 Oct 17;9:14925. doi: 10.1038/s41598-019-51096-x (PMC6797734; doi:10.1038/s41598-019-51096-x)
Supplement: Supplementary file 1 — Supplemental material [file 41598_2019_51096_MOESM1_ESM.docx]

Revisiting the identification of tumor sub-volumes predictive of residual uptake after (chemo)radiotherapy: influence of segmentation methods on ^18^F-FDG PET/CT images

Mathieu Hatt^1^, Florent Tixier^1,2^, Marie-Charlotte Desseroit^1,2^, Bogdan Badic^1^, Baptiste Laurent^1^, Dimitris Visvikis^1*^, Catherine Cheze Le Rest^1,2*^

^1^ LaTIM, INSERM, UMR 1101, Univ Brest, Brest, France.

^2^ Nuclear Medicine department, CHU Milétrie, Poitiers, France.

**Corresponding author:** Mathieu Hatt

* equally contributed

LaTIM INSERM UMR 1101

IBRBS – Institut Brestois de Recherche en Biologie et Santé

Faculté de médecine, 22 rue Camille Desmoulins, 29238 Brest, France

Tel: +33(0)2.98.01.81.11 E-mail: [hatt@univ-brest.fr](mailto:hatt@univ-brest.fr)

Supplemental material

Table 1: statistics of all overlap metrics and segmentations in the clinical cases

| Segmentation combinations / Metrics | Esophageal (n=9) | | | | H&N (n=10) | | | |
| --- | --- | --- | --- | --- | --- | --- | --- | --- |
|  | Dice | OF | X | Y | Dice | OF | X | Y |
| FLAB | 0.30±0.23  (0.28, 0.67, 0.02) | 0.42±0.23  (0.47, 0.70, 0.05) | 0.31±0.27  (0.21, 0.70, 0.01) | 0.37±0.21  (0.41, 0.66, 0.02) | 0.17±0.21  (0.09, 0.61, 0.00) | 0.27±0.30  (0.12, 0.70, 0.00) | 0.17±0.21  (0.08, 0.55, 0.00) | 0.23±0.29  (0.11, 0.70, 0.00) |
| 3040 | 0.41±0.24  (0.38, 0.75, 0.00) | 0.59±0.29  (0.62, 0.93, 0.00) | 0.35±0.23  (0.29, 0.66, 0.00) | 0.59±0.29  (0.62, 0.93, 0.00) | 0.32±0.18  (0.32, 0.65, 0.08) | 0.52±0.30  (0.52, 0.98, 0.13) | 0.28±0.20  (0.21, 0.66, 0.05) | 0.49±0.31  (0.43, 0.98, 0.13) |
| 4040 | 0.41±0.24  (0.35, 0.78, 0.00) | 0.56±0.28  (0.68, 0.88, 0.00) | 0.42±0.29  (0.31, 0.79, 0.00) | 0.50±0.28  (0.48, 0.88, 0.00) | 0.31±0.20  (0.31, 0.64, 0.07) | 0.49±0.30  (0.46, 0.91, 0.09) | 0.33±0.24  (0.28, 0.78, 0.04) | 0.41±0.30  (0.35, 0.91, 0.06) |
| 5040 | 0.38±0.22  (0.40, 0.74, 0.00) | 0.58±0.31  (0.72, 0.87, 0.00) | 0.47±0.32  (0.34, 0.87, 0.00) | 0.41±0.26  (0.37, 0.84, 0.00) | 0.29±0.20  (0.27, 0.60, 0.03) | 0.45±0.29  (0.45, 0.87, 0.06) | 0.35±0.27  (0.32, 0.87, 0.04) | 0.32±0.26  (0.28, 0.76, 0.02) |
| 6040 | 0.33±0.20  (0.38, 0.66, 0.00) | 0.59±0.33  (0.73, 0.93, 0.00) | 0.52±0.35  (0.38, 0.93, 0.00) | 0.31±0.24  (0.26, 0.73, 0.00) | 0.24±0.19  (0.24, 0.53, 0.02) | 0.39±0.28  (0.38, 0.94, 0.04) | 0.36±0.28  (0.32, 0.94, 0.04) | 0.22±0.19  (0.22, 0.60, 0.01) |
| 7040 | 0.27±0.18  (0.29, 0.52, 0.00) | 0.59±0.38  (0.59, 1.00, 0.00) | 0.55±0.39  (0.47, 1.00, 0.00) | 0.22±0.19  (0.18, 0.59, 0.00) | 0.18±0.15  (0.18, 0.45, 0.01) | 0.40±0.31  (0.35, 0.99, 0.03) | 0.40±0.31  (0.35, 0.99, 0.03) | 0.14±0.12  (0.13, 0.39, 0.00) |
| 8040 | 0.19±0.15  (0.20, 0.47, 0.00) | 0.60±0.40  (0.67, 1.00, 0.00) | 0.60±0.40  (0.67, 1.00, 0.00) | 0.13±0.13  (0.11, 0.39, 0.00) | 0.11±0.11  (0.09, 0.35, 0.00) | 0.43±0.33  (0.36, 1.00, 0.00) | 0.43±0.33  (0.36, 1.00, 0.00) | 0.07±0.08  (0.05, 0.25, 0.00) |
| 9040 | 0.07±0.07  (0.06, 0.20, 0.00) | 0.64±0.42  (0.71, 1.00, 0.00) | 0.64±0.42  (0.71, 1.00, 0.00) | 0.04±0.04  (0.03, 0.12, 0.00) | 0.04±0.05  (0.01, 0.16, 0.00) | 0.46±0.38  (0.44, 1.00, 0.00) | 0.46±0.38  (0.44, 1.00, 0.00) | 0.02±0.03  (0.01, 0.09, 0.00) |
| 3090 | 0.03±0.02  (0.02, 0.06, 0.00) | 0.86±0.34  (1.00, 1.00, 0.00) | 0.01±0.01  (0.01, 0.03, 0.00) | 0.86±0.34  (1.00, 1.00, 0.00) | 0.01±0.01  (0.01, 0.02, 0.00) | 0.55±0.44  (0.51, 1.00, 0.00) | 0.01±0.01  (0.00, 0.01, 0.00) | 0.55±0.44  (0.51, 1.00, 0.00) |
| 4090 | 0.04±0.04  (0.03, 0.09, 0.00) | 0.82±0.34  (1.00, 1.00, 0.00) | 0.02±0.02  (0.01, 0.05, 0.00) | 0.82±0.34  (1.00, 1.00, 0.00) | 0.01±0.02  (0.01, 0.04, 0.00) | 0.47±0.49  (0.34, 1.00, 0.00) | 0.01±0.01  (0.00, 0.02, 0.00) | 0.47±0.49  (0.34, 1.00, 0.00) |
| 5090 | 0.05±0.05  (0.04, 0.13, 0.00) | 0.73±0.43  (1.00, 1.00, 0.00) | 0.03±0.03  (0.02, 0.07, 0.00) | 0.73±0.43  (1.00, 1.00, 0.00) | 0.02±0.02  (0.01, 0.05, 0.00) | 0.44±0.50  (0.19, 1.00, 0.00) | 0.01±0.01  (0.00, 0.03, 0.00) | 0.44±0.50  (0.19, 1.00, 0.00) |
| 6090 | 0.07±0.08  (0.02, 0.20, 0.00) | 0.67±0.44  (1.00, 1.00, 0.00) | 0.04±0.05  (0.01, 0.12, 0.00) | 0.67±0.44  (1.00, 1.00, 0.00) | 0.02±0.03  (0.01, 0.08, 0.00) | 0.40±0.47  (0.12, 1.00, 0.00) | 0.01±0.02  (0.00, 0.04, 0.00) | 0.40±0.47  (0.12, 1.00, 0.00) |
| 7090 | 0.09±0.11  (0.03, 0.30, 0.00) | 0.53±0.45  (0.50, 1.00, 0.00) | 0.05±0.07  (0.01, 0.17, 0.00) | 0.53±0.45  (0.50, 1.00, 0.00) | 0.04±0.05  (0.01, 0.13, 0.01) | 0.35±0.44  (0.04, 1.00, 0.00) | 0.02±0.03  (0.00, 0.07, 0.00) | 0.35±0.44  (0.04, 1.00, 0.00) |
| 8090 | 0.13±0.17  (0.05, 0.44, 0.00) | 0.44±0.45  (0.50, 1.00, 0.00) | 0.09±0.12  (0.03, 0.31, 0.00) | 0.44±0.45  (0.50, 1.00, 0.00) | 0.04±0.07  (0.00, 0.17, 0.00) | 0.17±0.30  (0.00, 0.83, 0.00) | 0.02±0.04  (0.00, 0.10, 0.00) | 0.17±0.30  (0.00, 0.83, 0.00) |
| 9090 | 0.12±0.19  (0.00, 0.53, 0.00) | 0.22±0.35  (0.00, 1.00, 0.00) | 0.10±0.18  (0.00, 0.53, 0.00) | 0.22±0.35  (0.00, 1.00, 0.00) | 0.04±0.09  (0.00, 0.29, 0.00) | 0.08±0.17  (0.00, 0.50, 0.00) | 0.03±0.07  (0.00, 0.02, 0.00) | 0.08±0.17  (0.00, 0.50, 0.00) |
